# Clinical analysis of association between vaginal microbiota indicators and HPV infection

**DOI:** 10.3389/fgwh.2026.1809449

**Published:** 2026-03-23

**Authors:** Panpan Duan, Chengcheng Zhang, Qianqian Yang

**Affiliations:** 1 Department of Anesthesiology, Changhai Hospital of Naval Medical University, Shanghai, China; 2 Department of Obstetrics and Gynecology, Changhai Hospital of Naval Medical University, Shanghai, China

**Keywords:** HPV infection, risk factor, sialidase, vaginal cleanliness, vaginal microbiota

## Abstract

**Objective:**

To investigate the correlation between vaginal microbiota indicators and HPV infection, providing a reference for clinical prevention and treatment of HPV-related diseases.

**Methods:**

Clinical data of 5,362 female patients who underwent vaginal microbiota testing and HPV detection were retrospectively analyzed. The distribution of vaginal microbiota indicators and their association with HPV infection were statistically analyzed using the chi-square test. Three hundred fifty-five patients infected with HPV 16/18 were enrolled, followed up their HPV re-detection results at 12 months, and performed univariate analysis and logistic regression analysis to identify the risk factors for persistent HPV infection.

**Results:**

Among the 5,362 subjects, the positive rates of hydrogen peroxide, sialidase, leukocyte esterase, Trichomonas vaginalis, mold, and clue cells were 96.38%, 16.51%, 59.29%, 0.93%, 16.09%, and 8.65% respectively. 63.19% of the subjects had vaginal cleanliness of III-IV, and 71.73% had a vaginal pH value >4.5. Statistical analysis showed that sialidase (*χ*^2^ = 4.13, *P* = 0.04), and vaginal cleanliness (*χ*^2^ = 8.32, *P* = 0.00) were significantly associated with HPV infection; Vaginal Cleanliness(*χ*^2^ = 8.32, *P* = 0.04) and pH(*χ*^2^ = 14.63, *P* = 0.00) were significantly associated with HPV Genotypes. Vaginal pH value (OR = 2.65, 95% CI: 1.55–4.52, *P* < 0.001), N-acetylglucosaminidase positive (OR = 2.14, 95% CI: 1.12–4.08, *P* = 0.021), and fungus detected (OR = 1.88, 95% CI: 1.06–3.35, *P* = 0.032) were identified as independent risk factors for persistent HPV infection.

**Conclusion:**

Vaginal microbiota imbalance, as indicated by abnormal sialidase, poor vaginal cleanliness, and elevated pH value, may be related to HPV infection. Vaginal pH value, N-acetylglucosaminidase positive and fungus detected were identified as independent risk factors for persistent HPV infection.

## 1 Introduction

The vaginal microecology is a complex ecosystem composed of various microorganisms, which plays a crucial role in maintaining vaginal health (1). Imbalances in the vaginal microecology can lead to various gynecological diseases, such as vaginitis and cervicitis.

Human papillomavirus (HPV) infection is a major risk factor for cervical cancer and other genital tract diseases (2, 3). There are many factors that lead to HPV infection, such as: unhealthy sexual behavior, decreased individual immunity, age, and bad living habits (4, 5). Recent studies have suggested a potential association between vaginal microbiota imbalance and HPV infection, but the specific relationship remains unclear (6). Systematic analysis of the correlation between HPV infection and vaginal microecology not only holds important implications for clinical diagnosis and treatment, but also helps identify high-risk groups for cervical lesions, thereby providing guidance for the prevention and management of cervical lesions (7, 8).

This study retrospectively analyzed the clinical data of female patients who underwent vaginal microbiota testing and HPV detection, aiming to explore the correlation between various vaginal microbiota indicators and HPV infection, and provide a theoretical basis for the prevention and treatment of HPV-related diseases. Meanwhile, this study explored the risk factors and independent risk factors among vaginal microecological indicators associated with persistent HPV16/18 infection, so as to provide guidance for women with abnormal vaginal microecology complicated by HPV infection.

## 2 Materials and methods

### 2.1 Clinical data

A total of 5,362 female patients who underwent vaginal microbiota testing and HPV detection in Changhai hospital from 2024 to 01 to 2024-12 were included in this study. All subjects were placed in the lithotomy position during sampling. A vaginal speculum was used to fully expose the cervix and vagina, and a sterile cotton swab was dipped in vaginal secretions to collect test samples, which were then detected using the wet mount method supplemented by the staining method. After collecting the secretion samples, a cervical brush was used to scrape the surface of the cervix, the external orifice of the cervical canal, and the vaginal wall. Polymerase Chain Reaction (PCR) was then employed for HPV detection and genotyping. All subjects were informed and agreed to participate in the study, which was approved by the Ethics Committee of Changhai hospital (NO.CHEC2023-014).

### 2.2 Detection indicators and methods

Vaginal microbiota indicators: Hydrogen peroxide, sialidase, leukocyte esterase, Trichomonas vaginalis, cleanliness, mold, clue cells, and Potential of Hydrogen (pH) value were detected using routine vaginal secretion testing methods.

### 2.3 HPV detection

The results of HPV detection were classified into HPV 16/18, other high-risk HPV types, and low-risk HPV types.

### 2.4 Statistical analysis

SPSS 22.0 statistical software was used for data analysis. The count data were expressed as *N* (%), and the chi-square test was used to analyze the correlation between vaginal microbiota indicators and HPV infection. *P* < 0.05 was considered statistically significant. Logistic regression analysis was conducted to analyze the risk factors and independent risk factors for persistent HPV infection.

## 3 Results

### 3.1 Distribution of vaginal microbiota indicators

The distribution of vaginal microbiota indicators in 5,362 subjects is shown in Table 1. The positive rate of hydrogen peroxide was the highest (96.38%), followed by leukocyte esterase (59.29%). The positive rate of Trichomonas vaginalis was the lowest (0.93%). Regarding vaginal cleanliness, 63.19% of the subjects were classified as III-IV. The proportion of subjects with a pH value >4.5 was 71.73%.

**Table 1:** Distribution of vaginal microbiota indicators.

Indicators	Result	*N*/%
H2O2	+	5,168 (96.38%)
	-	194 (3.62%)
Sialidase	+	885 (16.51%)
	-	4,477 (83.49%)
Leukocyte esterase	+	3,179 (59.29%)
	-	2,183 (40.71%)
Trichomonas vaginalis	+	50 (0.93%)
	-	5,312 (99.07%)
Vaginal cleanliness	I-II	1,974 (36.81%)
	III-IV	3,388 (63.19%)
Fungus	+	863 (16.09%)
	-	4,499 (83.91%)
Clue cell	+	464 (8.65%)
	-	4,898 (91.35%)
HPV	16/18	355 (6.62%)
	Other high-risk HPV	903 (16.84%)
	Low-risk HPV	235 (4.38%)
	Negative	3,869 (72.16%)
pH	3.8–4.5	1,516 (28.27%)
	>4.5	3,846 (71.73%)

### 3.2 Correlation between vaginal microbiota indicators and HPV infection

As shown in Table 2, the overall HPV infection rate in the subjects was 27.87% (1,493/5,362). The results of the chi-square test showed that sialidase (*χ*^2^ = 4.13, *P* = 0.04), and vaginal cleanliness (*χ*^2^ = 8.32, *P* = 0.00) were significantly associated with HPV infection. There was no significant correlation between hydrogen peroxide, leukocyte esterase, Trichomonas vaginalis, mold, clue cells, pH and HPV infection (*P* > 0.05).

**Table 2:** Correlation between vaginal microbiota indicators and HPV infection.

Indicators	Result	HPV-positive	HPV-negative	*χ* ^2^	*P*
H_2_O_2_	+	1,442 (27.90%)	3,726 (72.10%)	3.54	0.06
	-	51 (26.29%)	143 (73.71%)		
Sialidase	+	264 (30.17%)	618 (69.83%)	4.13	0.04
	-	1,229 (27.38%)	3,251 (72.62%)		
Leukocyte esterase	+	877 (27.59%)	2,302 (72.41%)	0.26	0.61
	-	616 (28.22%)	1,567 (71.78%)		
Trichomonas vaginalis	+	13 (26.00%)	37 (74.00%)	0.75	0.39
	-	1,480 (27.86%)	3,832 (72.14%)		
Vaginal cleanliness	I-II	517 (26.19%)	1,457 (73.81%)	8.32	0.00
	III-IV	976 (28.81%)	2,412 (71.19%)		
Fungus	+	263 (30.48%)	600 (69.52%)	0.65	0.42
	-	1,230 (27.34%)	3,269 (72.66%)		
Clue cell	+	153 (33.01%)	311 (66.99%)	0.27	0.60
	-	1,340 (27.36%)	3,558 (72.64%)		
pH	3.8–4.5	440 (29.02%)	1,076 (70.98%)	2.17	0.14
	>4.5	1,053 (27.38%)	2,793 (72.62%)		

### 3.3 Correlation between vaginal microbiota indicators and HPV genotypes

Table 3 shows the correlation between vaginal microbiota indicators and different HPV genotypes. For vaginal cleanliness, the proportion of HPV 16/18 infection in subjects with cleanliness I-II was 6.89%, and that in subjects with cleanliness III-IV was 6.46% (*χ*^2^ = 8.32, *P* = 0.04). For pH value, the proportion of HPV 16/18 infection in subjects with pH 3.8–4.5 was 6.46%, and that in subjects with pH >4.5 was 5.25% (*χ*^2^ = 14.63, *P* = 0.00). There was no significant correlation between other vaginal microbiota indicators and HPV genotypes (*P* > 0.05).

**Table 3:** Correlation between vaginal microbiota indicators and HPV genotypes.

Indicators	Result	HPV				χ^2^	*P*
		16/18	Other high-risk HPV	Low-risk HPV	Negative		
H_2_O_2_	+	341 (6.60%)	878 (16.99%)	223 (4.31%)	3,726 (72.10%)	3.54	0.32
	-	14 (7.22%)	25 (12.89%)	12 (6.18%)	143 (73.71%)		
Sialidase	+	56 (6.33%)	169 (19.10%)	39 (4.41%)	618 (69.83%)	4.13	0.25
	-	299 (6.68%)	734 (16.39%)	196 (4.38%)	3,251 (72.62%)		
Leukocyte esterase	+	207 (6.51%)	543 (17.08%)	127 (3.09%)	2,302 (72.41%)	0.26	0.97
	-	148 (6.78%)	360 (16.49%)	108 (4.95%)	1,567		
Trichomonas vaginalis	+	3 (0.06%)	9 (0.17%)	1 (0.02%)	37 (0.70%)	0.75	0.86
	-	352 (6.63%)	894 (16.83%)	234 (4.41%)	3,832 (72.14%)		
Vaginal cleanliness	I-II	136 (6.89%)	310 (15.71%)	71 (3.60%)	1,457 (73.81%)	8.32	0.04
	III-IV	219 (6.46%)	593 (17.50%)	164 (4.84%)	2,412 (71.20%)		
Fungus	+	59 (6.84%)	156 (18.08%)	48 (5.56%)	600 (69.52%)	0.65	0.89
	-	355 (7.89%)	903 (20.07%)	235 (5.22%)	3,869 (85.99%)		
Clue cell	+	33 (7.11%)	96 (20.69%)	24 (5.17%)	311 (66.99%)	0.27	0.97
	-	322 (6.57%)	807 (16.43%)	211 (4.31%)	3,558 (72.64%)		
pH	3.8–4.5	98 (6.46%)	261 (17.22%)	81 (5.34%)	1,076 (70.98%)	14.63	0.00
	>4.5	257 (5.25%)	642 (13.10%)	154 (3.14%)	3,846 (71.73%)		

### 3.4 Univariate analysis of persistent HPV16/18 infection

The follow-up HPV test results of 355 patients with HPV16/18 infection at 12 months were compiled to assess the indicators associated with persistent HPV infection. Among these indicators, positive leukocyte esterase (OR = 1.95, 95% CI: 1.04–3.66, *P* = 0.038), positive N-acetylglucosaminidase (OR = 2.47, 95% CI: 1.22–4.99, *P* = 0.012), abnormal vaginal pH value (OR = 3.12, 95% CI: 1.89–5.15, *P* < 0.001), vaginal cleanliness grade III/IV (OR = 2.52, 95% CI: 1.02–6.24, *P* = 0.045) and fungal infection (OR = 2.91, 95% CI: 1.44–5.88, *P* < 0.003) were identified as risk factors for persistent HPV16/18 infection (Table 4).

**Table 4:** Univariate analysis of persistent HPV16/18 infection.

Indicators	OR	95% CI	*P*
H2O2 (+)	0.78	(0.32–1.91)	0.587
Sialidase (+)	1.48	(0.63–3.46)	0.372
Leukocyte esterase (+)	1.95	(1.04–3.66)	0.038
*β*-Glucuronidase (+)	1.43	(0.52–3.94)	0.489
N-Acetylglucosaminidase (+)	2.47	(1.22–4.99)	0.012
Abnormal PH	3.12	(1.89–5.15)	<0.001
Trichomonas vaginalis(+)	1.75	(0.32–9.52)	0.520
Vaginal cleanliness III/IV	2.52	(1.02–6.24)	0.045
Fungus (+)	2.91	(1.44–5.88)	0.003
Lactobacillus (+)	2.10	(0.67–6.55)	0.204
Clue cell (+)	1.38	(0.42–4.57)	0.595

### 3.5 Logistic regression analysis of vaginal microecological indicators and independent risk factors for persistent HPV infection

A further analysis of independent risk factors for persistent HPV infection was conducted. The results showed that vaginal pH value (OR = 2.65, 95% CI: 1.55–4.52, *P* < 0.001), N-acetylglucosaminidase positive (OR = 2.14, 95% CI: 1.12–4.08, *P* = 0.021), and fungus detected (OR = 1.88, 95% CI: 1.06–3.35, *P* = 0.032) were identified as independent risk factors for persistent HPV infection (Table 5).

**Table 5:** Logistic regression analysis of vaginal microecological indicators and independent risk factors for persistent HPV infection.

Indicators	OR	95% CI	*P*
Leukocyte esterase(+)	1.73	(0.94–3.18)	0.078
Vaginal cleanliness III/IV	1.92	(0.88–4.20)	0.102
Abnormal PH	2.65	(1.55–4.52)	<0.001
N-Acetylglucosaminidase (+)	2.14	(1.12–4.08)	0.021
Fungus (+)	1.88	(1.06–3.35)	0.032

## 4 Discussion

The vagina is a dynamic ecosystem, and its microbiota balance is crucial for maintaining vaginal health (9). This study analyzed the correlation between various vaginal microbiota indicators and HPV infection, and the results showed that sialidase, vaginal cleanliness, and pH value were significantly associated with HPV infection.

Sialidase is an enzyme produced by some anaerobic bacteria, and its positive expression indicates the presence of bacterial vaginosis (10, 11). In this study, the positive rate of sialidase in HPV-infected subjects was 30.17%, which was significantly higher than that in HPV-negative subjects (27.38%), suggesting that bacterial vaginosis may increase the risk of HPV infection. This is consistent with previous studies that have shown that bacterial vaginosis can disrupt the vaginal microenvironment, reduce local immunity, and facilitate HPV invasion and persistence (12). Vaginal cleanliness is an important indicator reflecting the vaginal microenvironment and may related to group B streptococcus infection (13). This study found that the proportion of HPV infection in subjects with cleanliness III-IV (28.81%) was higher than that in subjects with cleanliness I-II (26.19%), indicating that poor vaginal cleanliness is associated with HPV infection. The possible reason is that the increase in pathogenic bacteria and the decrease in lactobacilli in the vagina with poor cleanliness lead to the destruction of the vaginal barrier function, making it easier for HPV to infect. The pH value of the vagina is mainly maintained by lactobacilli through the production of lactic acid. A normal vaginal pH value (3.8–4.5) is conducive to inhibiting the growth of pathogenic microorganisms (14). In this study, the proportion of subjects with a pH value >4.5 was 71.73%, and the HPV infection rate in this group was 27.38%. Although the difference was not statistically significant in the overall analysis, the correlation with HPV genotypes showed a significant difference, suggesting that an increase in vaginal pH value may be related to HPV infection (15). This may be because the increase in pH value reduces the number and activity of lactobacilli, weakens the vaginal defense function, and increases the susceptibility to HPV (16). Studies have indicated that women with a normal vaginal microecology inherently possess a stronger ability to clear HPV (17). Furthermore, an abnormal vaginal microecology can increase the risk of high-grade cervical lesions. They also emphasize the significant importance of maintaining a healthy vaginal microecology in preventing cervical lesions (18, 19).

In addition, this study found no significant correlation between hydrogen peroxide, leukocyte esterase, Trichomonas vaginalis, mold, clue cells and HPV infection, which may be related to the small sample size or the differences in detection methods. Hydrogen peroxide is a marker of lactobacilli activity, and its negative expression indicates a decrease in lactobacilli (20). However, in this study, only 3.62% of the subjects were hydrogen peroxide negative, which may affect the statistical results.

Patients with low immunity or persistent chronic vaginal inflammation may have an increased risk of HPV infection, and persistent HPV16/18 infection is a major cause of cervical cancer. Given the high oncogenic risk of HPV16/18 in cervical cancer, colposcopy and cervical biopsy are recommended at initial detection to rule out cervical lesions, even if cervical cytology results are normal. Regular follow-up is also required during long-term management to avoid missing persistent HPV16/18 infection (21).The present study found that positive leukocyte esterase, positive N-acetylglucosaminidase, abnormal vaginal pH value, vaginal cleanliness grade III/IV, and fungal infection were identified as risk factors for persistent HPV16/18 infection. Furthermore, abnormal vaginal pH value, positive N-acetylglucosaminidase, and fungal detection were independent risk factors for persistent HPV infection, highlighting the importance of vaginal microecological management. The vagina normally maintains an acidic environment, and its pH value directly reflects changes in the vaginal microenvironment. Patients with abnormal vaginal pH carry an elevated risk of HPV infection (22). N-acetylglucosaminidase is secreted by microorganisms such as Candida species and Trichomonas vaginalis. Abnormal levels of this enzyme indicate damage to the vaginal and cervical mucosal barrier, which increases the risk of HPV colonization and persistent infection. These findings also support that positive fungal test results are an independent risk factor for persistent HPV16/18 infection (23).Therefore, sufficient attention should be paid to patients with newly detected HPV16/18 infection combined with the above abnormal indicators. The follow-up interval should be shortened and early intervention implemented to prevent the progression of cervical lesions.

This study has some limitations. First, it is a retrospective study, and there may be selection bias. Second,other factors that may affect HPV infection, such as sexual behavior and immune status, were not considered. Future studies with larger sample sizes and prospective designs are needed to further verify the relationship between vaginal microbiota and HPV infection.

## 5 Conclusions

Vaginal microbiota imbalance, as indicated by abnormal sialidase, poor vaginal cleanliness, and elevated pH value, is associated with HPV infection. Vaginal pH value, N-acetylglucosaminidase positive and fungus detected were identified as independent risk factors for persistent HPV infection. Monitoring and regulating vaginal microbiota may be helpful for the prevention and treatment of HPV-related diseases.

## Data Availability

The original contributions presented in the study are included in the article/Supplementary Material, further inquiries can be directed to the corresponding author.
